# Applying the Nova food classification to food product databases using discriminative ingredients: a methodological proposal

**DOI:** 10.3389/fpubh.2025.1575136

**Published:** 2025-07-01

**Authors:** Mariana Fagundes Grilo, Beatriz Silva Nunes, Ana Clara Duran, Camila Zancheta Ricardo, Larissa Galastri Baraldi, Euridice Martinez Steele, Camila Aparecida Borges

**Affiliations:** ^1^Center for Food Studies and Research, University of Campinas, Campinas, Brazil; ^2^Department of Exercise and Nutrition Sciences, The George Washington University, Washington, DC, United States; ^3^Graduate Program in Collective Health, Faculty of Medical Sciences, University of Campinas, Campinas, Brazil; ^4^Center for Epidemiological Studies in Nutrition and Health, University of São Paulo, São Paulo, Brazil; ^5^Center for Research in Food Environments and Prevention of Chronic Diseases Associated with Nutrition (CIAPEC), Institute of Nutrition and Food Technology (INTA), University of Chile, Santiago, Chile; ^6^Department of Food Science and Technology, University of São Paulo, São Paulo, Brazil

**Keywords:** ultra-processed foods, methodology, classification, food substances, food additives

## Abstract

**Background:**

Growing interest in the Nova food classification system surged among various stakeholders, driven primarily by compelling evidence linking the consumption of ultra-processed foods (UPFs) to negative health outcomes. This growing interest underscores the potential value of identifying clear markers to classify UPFs, particularly to support research and regulatory efforts.

**Objective:**

To propose replicable methods to identify UPFs, by testing the sensitivity and specificity of these methods using a large sample of packaged foods from the 2017 Brazilian Food Labels Database.

**Methods:**

We created five scenarios to identify UPFs using substances of rare culinary use and food additives typically found in UPFs and compared them with the Nova food classification process based on the product name and food categories, considered the classic method to identify UPFs. We estimated the proportion of foods and beverages identified as UPFs using the different scenarios based on the presence of discriminative ingredients. We used a diagnostic test and a receiver operating characteristic (ROC) to understand which of the five scenarios performed better compared to the classic method to identify UPFs. Finally, we conducted a sensitivity analysis to test the role of vitamins and minerals in identifying UPFs.

**Results:**

We found variations in UPFs prevalence from 47 to 72% across the five scenarios, compared to 70% using the classic method to identify UPFs in Brazilian packaged foods. The scenario using food additives of a sole cosmetic function and substances of rare culinary use (scenario 3) identified a 65% UPF, while maintaining reasonable sensitivity and specificity, and the best-performing ROC curve. There was no significant difference in identifying UPFs when comparing the addition of vitamins and minerals to the food additives with sole cosmetic function.

**Conclusion:**

This study shows that using ingredient-based criteria, specifically cosmetic additives and substances of rare culinary use, can reliably identify UPFs, offering reproducibility, and supporting its use in research and policy applications.

## 1 Introduction

Over the past two decades, the Nova food classification system has transformed dietary assessment research by shifting the focus from nutrient-based analyses to consideration of the extent and purpose of industrial processing of foods and beverages (1). This system classifies foods into 4 major groups: unprocessed and minimally processed foods, processed culinary ingredients, processed foods, and ultra-processed foods (UPFs). The latter are defined as industrial formulations, typically made of food components that have been either modified or recombined, with little or no whole foods, as well as industrial substances and food additives aimed at increasing durability and enhancing or modifying sensory characteristics such as color, taste, odor and texture of foods (2).

Over the years, the Nova food classification has proved associated higher consumption of UPFs with adverse health outcomes, including weight gain (3), type 2 diabetes (4), cardiometabolic diseases (5), cerebrovascular disease (5), cancer (6), premature deaths (7), and all-cause mortality (5). The Nova food classification has proven valuable for monitoring dietary patterns in predicting the nutritional quality of diets and in identifying the increasing consumption of UPFs over time in diverse populations around the world (8–12) and has been widely used. The Nova food classification has been applied to observational studies (13, 14), cohort studies (15–17), and randomized trials (18) to assess the link between UPFs consumption and health outcomes.

However, despite the widespread adoption of Nova, its implementation in both research and policy remains largely reliant on product names, descriptions, or broad food categories rather than on objective, reproducible criteria grounded in product characteristics. While many governments, international agencies and academic groups have been implementing or proposing policies such as mandatory front-of-pack nutrition labeling regulations, marketing restrictions, and taxation policies that can consider the level of processing (19–21), there is growing interest in developing a more standardized and replicable approach.

The Nova food classification system itself describes two ingredient groups that are exclusively found in UPFs: substances of rare culinary use (e.g., hydrolyzed proteins, interesterified oils, and protein isolates) and food additives with cosmetic function (e.g., non-sugar sweeteners, flavor enhancers, colors, emulsifiers, thickeners, and anti-foaming agents). Still, only a limited number of studies have explored the utility of these markers for developing ingredient-level methods to identify UPFs (22, 23). As emphasized by Popkin et al. (22), there is an urgent need for more robust criteria grounded in ingredient-level markers to distinguish UPFs, what would enhance classification precision, support regulatory decision-making, and ultimately contribute to the development of more effective public health policies.

This study addresses this gap by evaluating the performance of different methods for identifying UPFs, combining the use of substances of rare culinary use and food additives with cosmetics function in a large database of products sold in Brazil.

## 2 Materials and methods

### 2.1 Data source

In this cross-sectional study, we used data from the 2017 Brazilian Food Labels Database (24). This database is among the most comprehensive resources with detailed information on the food composition of packaged foods sold in Brazil, with primary data collection (24). It includes the food products sold by the top five food retailers in Brazil, which collectively hold 69.7% of the national grocery retail market share (25). More detailed information on how the data were collected is available elsewhere (24). The database has been used in other nutrition-related studies (26, 27).

The 2017 Brazilian Food Labels Database contains 11,434 foods and beverages. Because we wanted to use the list of ingredients to classify foods and beverages according to the Nova food classification, foods and beverages that did not provide the list of ingredients (*n* = 1,574) on their package were excluded, totaling 9,860 unique products. It is important to note that in the Brazilian legislation, products that contain only one ingredient are exempted to show the list of ingredients on the package, i.e., in this case, the products that were excluded were fresh (e.g., rice beans, fresh fruits and vegetables) or culinary ingredient with only one ingredient (e.g., sugar, salt, oil) (28).

### 2.2 Scenarios to identify ultra-processed foods

For the purpose of the study, we created five scenarios to identify UPFs, according to the latest Nova food classification published in 2019, based mainly on the presence of food substances never or rarely used in kitchens, and food additives. Although excessive amounts of added sugar, fat, and sodium are often present in UPFs, the study focused on the presence of ingredients that are exclusive to UPFs, namely, food substances not commonly used in culinary preparations and cosmetic additives, as they represent the core criteria used to distinguish UPFs from other food groups in the Nova classification.

The food substances never or rarely used in kitchens (hereafter referred to as substances of rare culinary use, i.e., any material that is produced as a secondary result during the manufacturing or production process of food items), including high-fructose corn syrup, hydrogenated or interesterified oils, and hydrolyzed proteins (2). We also included added sugars, carbohydrates, modified oils, protein and fiber sources based on the definitions proposed by Zancheta et al. (2) (Supplementary Table 3).

To identify and classify food additives we used the information available in the 2023 Codex Alimentarius (29). The names of food additives were translated from English to Portuguese according to the National Health Surveillance Agency (ANVISA) standards, to align with the database language (30). Because food additives can have more than one function, we identified and distinguished food additives with sole a cosmetic function from food additives that could also have other functions. We excluded vitamins and minerals that can be used as food coloring from the search but used them in exploratory analysis to be described (Supplementary Table 5).

In agreement with the latest proposed definition of UPFs (1), we considered food additives with cosmetic functions the following: flavors, flavor enhancers, food colorings, emulsifiers, emulsifying salts, sweeteners, thickeners, and anti-foaming, bulking, carbonating, foaming, gelling and glazing agents (Supplementary Table 1). Besides looking for food additives that serve a cosmetic function in the list of ingredients, we also checked for non-technical terms for flavorings, such as ‘natural flavoring,’ as referenced in Brazilian legislation (RDC No. 2) (Supplementary Table 2). According to this regulation, it is not required to list each specific substance that makes up the flavorings on the ingredient list. Instead, the label can use general terms such as ‘natural flavorings’. The five proposed scenarios, detailed in Table 1, are described below:

**Table 1:** Scenarios for ultra-processed food classification.

Scenario	Key criterion
Scenario 1	Food substances of rare culinary use
Scenario 2	Food additives with sole cosmetic function
Scenario 3	Substances of rare culinary use and/or food additives with sole cosmetic function
Scenario 4	Food additive that can serve as an additive of cosmetic function
Scenario 5	Food substances of rare culinary use and/or food additive that can serve as an additive of cosmetic function
Classic method	Food names and categories

#### 2.2.1 Scenario 1: presence of at least one substance of rare culinary use

This scenario identifies UPFs based solely on the presence of substances of rare culinary use. Scenario 1 was included as a first analytical step to explore the potential of these substances as early markers of UPFs. This approach allows us to examine how individual ingredient groups contribute to UPF classification when applied progressively.

#### 2.2.2 Scenario 2: presence of at least one food additive with sole cosmetic function

Scenario 2 identifies UPFs based solely on the presence of food additives with cosmetic function. Recent studies, such as Zancheta et al. (31), underscore their significance as markers of ultra-processing, aligning with the Nova classification’s emphasis on the purpose of processing (2). This scenario is particularly relevant for policy initiatives, such as front-of-pack labeling, that aim to regulate products based on sensory-enhancing additives.

#### 2.2.3 Scenario 3: presence of at least one substance of rare culinary use and/or a food additive with sole cosmetic function

Scenario 3 identifies UPFs based on the presence of substances of rare culinary use and/or a food additive with sole cosmetic additive, recognizing their complementary roles in the industrial processing of UPFs. The rationale for this scenario is grounded on the definition provided by the developers of the Nova food classification for identifying UPFs through the evaluation of ingredient combinations. This approach considers both the nature of the ingredients, which are rarely used in culinary practices, and the intended purpose of food additives, which is to modify the sensory characteristics of food products, standardizing the ultra-processing construct (2).

#### 2.2.4 Scenario 4: presence of at least one food additive that can serve as an additive with cosmetic function

Scenario 4 explores the multifunctional roles of food additives, acknowledging that many additives, while officially claimed to serve one purpose, may also fulfill other functions that align with ultra-processing characteristics. For instance, food additives listed as preservatives may simultaneously enhance sensory attributes like texture or color, complicating their classification as UPF markers (2, 31). For example, sodium nitrite, sulfur dioxide, and sodium benzoate, in addition to their preservative roles, can also affect color, appearance, or flavor (29). This scenario broadens the scope to include these multifunctional food additives, aiming to assess whether their inclusion impacts the identification of UPFs compared to more narrowly defined criteria.

#### 2.2.5 Scenario 5: presence of at least one substance of rare culinary use and/or a food additive that can serve as an additive of cosmetic function

Scenario 5 adopts the most inclusive approach, combining all potential indicators of ultra-processing, including food substances of rare culinary use and/or food additive that can serve as an additive of cosmetic function.

### 2.3 Classic method: identification of ultra-processed foods using the food description

The classic method for identifying UPFs relies on food names and categories to classify products. This approach is widely used in dietary intake studies, such as studies using the Brazil’s Household Budget Survey (POF) and international surveys like NHANES (9, 32). While it enables population-level consumption assessment, it may not reflect specific product characteristics such as the presence of cosmetic additives or substances of rare culinary use. In this study, the classic method is used as a ‘gold standard’ to evaluate the performance of alternative scenarios based on ingredient composition.

All the discriminative ingredient terms used to identify food substances of rare culinary use and food additives in the five proposed scenarios are available in Supplementary Table 4.

### 2.4 Statistical analysis

We estimated the prevalence of UPFs in the Brazilian Food Labels Database using the five proposed scenarios and the classic method to identify UPFs, overall and by food category (31). Subsequently, we conducted diagnostic tests to assess the sensitivity, specificity, positive predictive value, and negative predictive value for each scenario in comparison with the classic method to identify UPFs. Then, we developed the receiver operating characteristic (ROC) curve to evaluate the performance of the scenarios. The ROC curve is a graphical representation of a binary classifier’s performance, plotted by sensitivity (true positive rate) against 1 – specificity (false positive rate). Its effectiveness is primarily gauged by the Area Under the Curve (AUC), with values closer to 1 indicating better performance. A superior model’s ROC curve approaches the top-left corner, reflecting a high sensitivity without a significant increase in false positives. The curve’s initial steepness and its concave shape towards the top-left are also signs of a robust classifier, indicating an effective balance between sensitivity and specificity. In essence, the closer and more bowed the curve towards the top-left, the better the model is at distinguishing between the two classes.

Finally, in the sensitivity analysis, we assessed the proportion of UPFs identified, including vitamins and minerals, in scenarios 2 and 3. We compared these proportions among the scenarios using a proportion test and repeated the diagnostic test (sensitivity, specificity, positive predictive value, and negative predictive value).

Analyses were performed with Stata/MP 16.1, College Station, TX: StataCorp LLC.

## 3 Results

**Figure 1:**
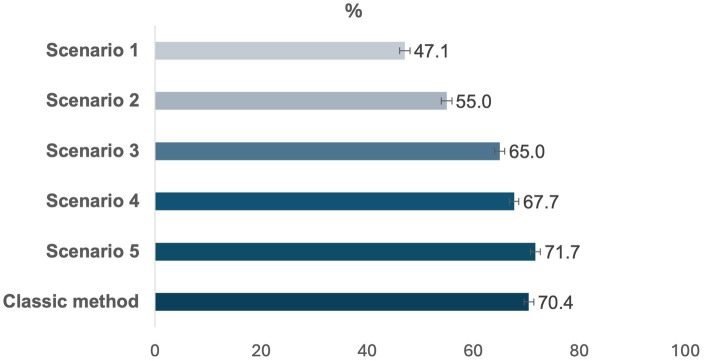
Prevalence of ultra-processed foods in the Brazilian food supply across the five proposed scenarios and the classic method to identify ultra-processed foods (UPFs) Brazilian Food Labels Database, 2017. 2. *Scenario 1: Presence of at least one food byproduct; Scenario 2: Presence of at least one food additive with sole cosmetic function; Scenario 3: Presence of at least one food byproduct or a food additive with sole cosmetic function; Scenario 4: Presence of at least one food additive that can serve as an additive with cosmetic function; Scenario 5: Presence of at least one food byproduct or a food additive that can serve as an additive of cosmetic function; ‘Classic method’ to identify UPF: Frequently used method to identify UPF by food names and food categories. **Bars: 95% confidence intervals.

The prevalence of UPFs in the Brazilian food supply across the five scenarios ranged from 47.1% (95% confidence interval, CI: 46.1–48.1) in scenario 1 to 71.7% (95% CI: 70.8–72.6) in scenario 5, compared with 70.5% (95% CI: 69.6–71.3) using the classic method to identify UPFs (Figure 1).

Table 2 shows that some food categories show consistently low prevalence of UPFs across all the scenarios. Canned vegetables (e.g., canned corn with salt and sugar, canned peas in brine) have a prevalence of 2.3% in Scenario 1, increasing to 13.9% in Scenario 5, with none of the products classified as UPFs under the classic method. Packaged fruits and vegetables (e.g., pre-cut pineapple with preservative, vacuum-packed cooked beets) show no or low (1.3%) prevalence of UPFs across all scenarios. Nuts and seeds (e.g., cashews, sunflower seeds) have 11.1 and 16.7% of products classified as UPFs under both Scenario 1 and Scenario 5, respectively, and 11% under the classic method (Table 2).

**Table 2:** Prevalence of ultra-processed foods (UPFs) by scenarios of UPFs identification by food category.

Food category	Total	Ultra-processed food identification											
		Scenario 1		Scenario 2		Scenario 3		Scenario 4		Scenario 5		Classic method	
		*n*	%	*n*	%	*n*	%	*n*	%	*n*	%	*n*	%
Breakfast cereals and granola bars	308	233	75.6	230	74.7	270	87.7	285	92.5	294	95.5	308	100.0
Bakery products	594	431	72.6	304	51.2	486	81.8	451	75.9	509	85.7	594	100.0
Convenience foods	795	390	49.1	447	56.2	549	69.1	514	64.7	586	73.7	795	100.0
Unsweetened dairy products	174	6	3.4	0	0.0	6	3.4	49	28.2	50	28.7	2	1.1
Sweetened dairy products	483	459	95.0	432	89.4	476	98.6	465	96.3	476	98.6	483	100.0
Salty snacks	356	140	39.3	239	67.1	257	72.2	261	73.3	272	76.4	346	97.2
Cookies	747	524	70.1	589	78.8	660	88.4	665	89.0	682	91.3	747	100.0
Canned vegetables	345	8	2.3	25	7.2	29	8.4	44	12.8	48	13.9	0	0.0
Oils and fats	294	23	7.8	68	23.1	73	24.8	100	34.0	103	35.0	46	15.6
Sauces and dressings	791	286	36.2	427	54.0	489	61.8	500	63.2	542	68.5	751	94.9
Coffee and tea	68	0	0.0	30	44.1	30	44.1	30	44.1	30	44.1	5	7.4
Candies and desserts	1,218	960	78.8	998	81.9	1,144	93.9	1,108	91.0	1,167	95.8	1,210	99.3
Cereals, beans, other grain products	463	25	5.4	73	15.8	91	19.7	108	23.3	119	25.7	62	13.4
Packaged fruits and vegetables	299	0	0.0	4	1.3	4	1.3	4	1.3	4	1.3	0	0.0
Meat, poultry, seafood, and egg	49	0	0.0	0	0.0	0	0.0	3	6.1	3	6.1	0	0.0
Sugar and other non-caloric sweeteners	66	14	21.2	37	56.1	41	62.1	42	63.6	42	63.6	44	66.7
Processed meats	810	381	47.0	530	65.4	572	70.6	577	71.2	594	73.3	617	76.2
Juices	144	41	28.5	34	23.6	55	38.2	36	25.0	55	38.2	46	31.9
Nectars	160	92	57.5	131	81.9	137	85.6	140	87.5	143	89.4	160	100.0
Fruit-flavored drinks	220	138	62.7	214	97.3	216	98.2	217	98.6	218	99.1	220	100.0
Sodas	106	26	24.5	104	98.1	104	98.1	105	99.1	105	99.1	106	100.0
Other beverages	286	141	49.3	219	76.6	238	83.2	249	87.1	259	90.6	269	94.1
Nuts and seeds	72	8	11.1	8	11.1	12	16.7	8	11.1	12	16.7	8	11.1
Cheeses	607	109	18.0	174	28.7	241	39.7	500	82.4	509	83.9	126	20.8
Fruit preserve	405	208	51.4	104	25.7	231	57.0	214	52.8	248	61.2	1	0.2

Brazilian Food Labels Database, 2017.

*Scenario 1: Presence of at least one substances of rare culinary use; Scenario 2: Presence of at least one food additive with sole cosmetic function; Scenario 3: Presence of at least one substances of rare culinary use and/or a food additive with sole cosmetic function; Scenario 4: Presence of at least one food additive that can serve as an additive with cosmetic function; Scenario 5: Presence of at least one substances of rare culinary use or a food additive that can serve as an additive of cosmetic function; Classic method to identify UPF: Frequently used method to identify UPFs by food names and food categories.

Some food categories exhibit a prevalence of UPFs greater than 60% across all methods. Breakfast cereals and granola bars (e.g., strawberry yogurt-flavored cereal bars) have a prevalence of 75.6% in Scenario 1, rising to 95.5% in Scenario 5, and reaching 100% in the classic method. Sweetened dairy products (e.g., fermented dairy drink; flavored Greek yogurt; yogurt with fruit preparation) show an UPFs prevalence of 95.0% in Scenario 1, 98.6% in Scenario 5, and 100% in the classic method. Fruit-flavored drinks (e.g., concentrated orange juice; pineapple-flavored drink powder; liquid fruit-flavored drink concentrate) are classified as UPFs in 62.7% of products under Scenario 1, rising to 99.1% in Scenario 5, and 100% in the classic method (Table 2).

Other food category that has low variability across scenarios include juices with 28.5% UPFs prevalence in Scenario 1, 38.2% in Scenario 5, and 31.9% under the classic method. In contrast, some food categories show higher variability in the prevalence of UPFs between Scenario 1, Scenario 5, and the classic method. For instance, unsweetened dairy products (e.g., skim yogurt; instant whole milk powder) are classified as UPFs in 3.4% of cases under Scenario 1, increases to 28.7% in Scenario 5, while the classic method classifies 1.1% as UPF. Similarly, cheeses (e.g., mozzarella cheese; cream cheese spread) show variability, with 18.0% classified as UPFs in Scenario 1, rising to 83.9% in Scenario 5, and 20.8% under the classic method. Convenience foods (e.g., frozen pizza, instant noodles) show an increase trend across scenarios, with 49.1% classified as UPFs in Scenario 1, 73.7% in Scenario 5, and 100% in the classic method, like salty snacks, with 39.3% classified as UPFs in Scenario 1, 76.4% in Scenario 5, and nearly all (97.2%) under the classic method. Then, fruit preserves (e.g., strawberry jam, guava paste) show different values, with 51.4% classified as UPFs in Scenario 1, rising to 61.2% in Scenario 5, but dropping to just 0.2% in the classic method, as well as coffee and tea (e.g., coffee beans or instant coffee, dried herbs for tea), not identified as UPF in Scenario 1, with a prevalence 44.1% being considered UPF in Scenario 5 and 7.4% in the classic method (Table 2).

Using a diagnostic test to compare the five scenarios of UPFs identification with the classic method to identify UPFs, we found increased sensitivity from scenario 1 (61.8%) to scenario 5 (86.9%), and decreased specificity (from 87.9% in scenario 1 to 64.5% in scenario 5) (Table 3).

**Table 3:** Diagnostic tests comparing the identification of ultra-processed foods (UPFs) using the five scenarios and Classic method.

Statistical metrics	Scenario 1	Scenario 2	Scenario 3	Scenario 4	Scenario 5
Sensitivity	61.8%	71.3%	82.6%	82.3%	86.9%
Specificity	87.9%	83.9%	76.8%	67.2%	64.5%
Positive predictive value	92.4%	91.3%	89.4%	85.7%	85.4%
Negative predictive value	49.1%	55.1%	64.9%	61.5%	67.4%

Brazilian Food Labels Database, 2017.

*Scenario 1: Presence of at least one substances of rare culinary use; Scenario 2: Presence of at least one food additive with sole cosmetic function; Scenario 3: Presence of at least one substances of rare culinary use and/or a food additive with sole cosmetic function; Scenario 4: Presence of at least one food additive that can serve as an additive with cosmetic function; Scenario 5: Presence of at least one substances of rare culinary use and/or a food additive that can serve as an additive of cosmetic function; Classic method to identify UPFs: Frequently used method to identify UPFs by food names and food categories.

**Figure 2:**
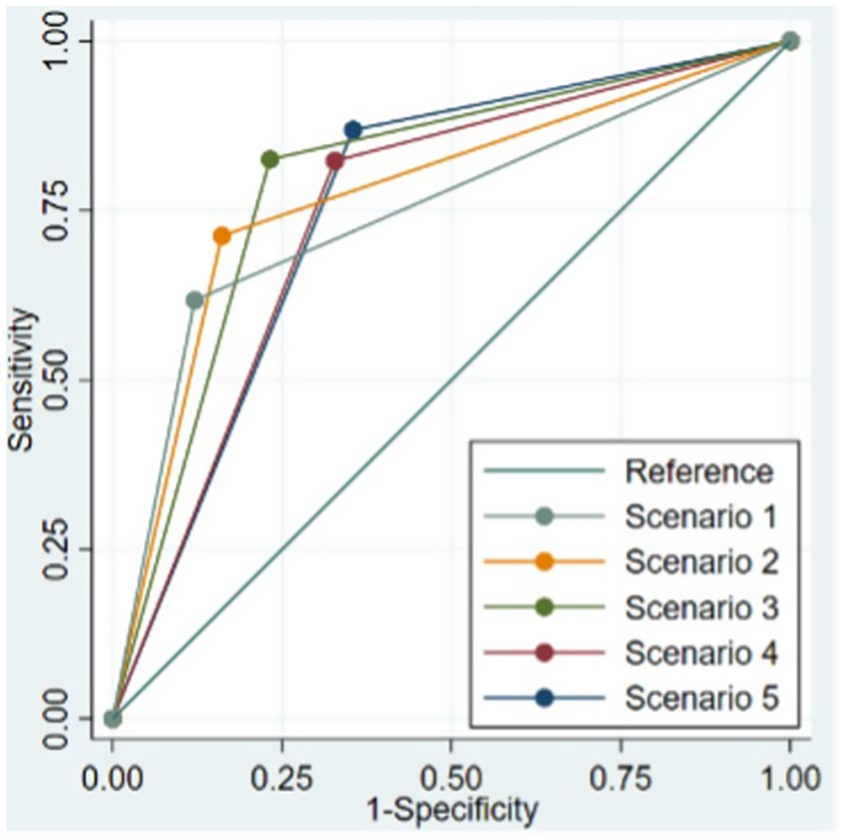
Receiver operating characteristic (ROC) curve for the five proposed scenarios having the classic method to identify ultra-processed foods (UPFs) as the reference. *Scenario 1: Presence of at least one food byproduct; Scenario 2: Presence of at least one food additive with sole cosmetic function; Scenario 3: Presence of at least one food byproduct or a food additive with sole cosmetic function; Scenario 4: Presence of at least one food additive that can serve as an additive with cosmetic function; Scenario 5: Presence of at least one food byproduct or a food additive that can serve as an additive of cosmetic function; ‘Classic method’ to identify UPF: Frequently used method to identify UPF by food names and food categories.

The area under the curve (AUC) for scenarios 1, 2, 3, 4, and 5 was 0.748, 0.776, 0.797, 0.748, and 0.757, respectively. Scenario 3, that captured 65% of the products as UPFs (95% CI: 64.1–66.0), had the highest AUC, indicating better performance compared to the classic method used to identify UPFs (Figure 2).

In the sensitivity analysis, the addition of vitamins and minerals that can serve as food additives with a cosmetic function showed no statistical differences compared with the analogous scenarios for which they were not included (scenarios 2 and 3) (Supplementary Table 6).

## 4 Discussion

In this study, we found that the choice of food classification method can significantly influence the estimated prevalence of UPFs in the Brazilian food supply. Across five different scenarios using UPF markers, the prevalence rates varied by approximately 25 percentage points compared to a 70% prevalence observed with the widely used classic method, which relies on food names and categories. Among the tested approaches, Scenario 3, which incorporates the presence of either substances of rare culinary use or food additives with sole cosmetic functions, stood out for effectively identifying a high proportion of UPFs. This scenario also demonstrated satisfactory classification performance when evaluated against the ROC curve, aligning closely with the results of the classic method.

The scenario based solely on the presence of substances of rare culinary use ensures high specificity by focusing on a well-defined characteristic of UPFs. However, it underestimates UPF prevalence by excluding other critical markers, such as food additives. Other studies in Brazil and in the U. S. (22, 23, 31) emphasize the importance of food additives as markers of ultra-processing, aligning with the Nova classification’s emphasis on the purpose of processing. Scenario 3 addresses this limitation by identifying UPFs based on the presence of substances of rare culinary use and/or food additives with solely cosmetic functions, recognizing their complementary roles in industrial processing. This approach balances specificity and sensibility, aligning more closely with the classic method for UPF identification.

An illustrative example of the strengths of Scenario 3 can be seen in its application to dairy products. In a study conducted in Chile by Zancheta et al. (31), approximately 30% of dairy products were classified as UPFs when all potential cosmetic additives, regardless of their sole cosmetic function, were included. In contrast, Scenario 3 in the current study, which focused only on food additives with sole cosmetic functions, showed a proportion of 3%.

Complementing these findings, Zancheta et al. (31) applied three scenarios to assess how different UPF classification methods influenced health outcomes: (i) classic Nova approach based on food names and categories; (ii) ingredient-based approach including all additives with potential cosmetic functions; and (iii) approach including only additives with solely cosmetic functions. The findings showed that the prevalence of UPFs and their associations with health outcomes, particularly measures of adiposity such as BMI and waist circumference, varied depending on the scenario used. For example, stronger associations with adiposity indicators were observed when broader additive criteria (i.e., including all potential cosmetic additives) were used (31).

The comparison between scenarios could aid future studies employing the Nova classification and assist in shaping public policies that need to distinguish UPFs from other foods and beverages. In view of the urgency of having standardized and replicable methods to identify UPFs, other studies have used the list of ingredients with this purpose, especially to assess UPFs consumption (31), and more recently to identify the presence of nutrients of public health concern in the food supply (22, 23). The reliance on discriminatory ingredients, such as a combination of substances of rare culinary use and/or food additives, introduces a level of detail and complexity in the classification process that adds to the literature on how to best operationalize the UPFs construct to be used in policies that require no or very low uncertainty in the definition of UPFs. The fact that Scenario 3 aligned more closely with the classic method to identify UPFs suggests that this scenario may provide more accurate UPFs estimates.

Although the list of ingredients does not provide information about the food matrix, using discriminatory ingredients that correspond to substances of rare culinary use and food additives with the function of altering the food or beverage’s physical characteristic allows for a more systematized method to identify UPFs (2), and can help evaluators to reach a consensus on the identification of these foods (33).

The findings showed that certain categories are more likely to consist solely of UPFs, like breakfast cereals and granola, sweetened dairy products, and fruit-flavored drinks. A different study using this same Brazilian food and beverage database found that 100% of sweet cookies, savory biscuits, margarine, cakes, sweet pies, chocolate, dairy beverages, and ice cream contained at least one cosmetic additive or exceeded recommended levels of critical nutrients like sugars, salt, oils, and fats. These results collectively emphasize the ubiquity of ultra-processing markers, such as cosmetic additives or excessive nutrients, in certain product groups (23). For other food categories, such as coffee and tea, and fruit preserve, there were discrepancies between scenarios. They could be considered fresh or minimally processed/processed by name and food group, but they may contain markers of ultra-processing in the list of ingredients, such as food additives with cosmetic function.

Regarding the trade-off between sensitivity and specificity in the diagnostic test comparing all the five scenarios with the classic method to identify UPFs, while an increase in sensitivity from scenario 1 to scenario 5 is promising in accurately identifying true positive cases (decreasing the false negative UPFs), there is an expected decrease in specificity, due to increasing false positive UPFs. We highlight that even with a more conservative approach to identify UPFs, scenario 3 demonstrated a sensitivity of 82.6% and a specificity of 76.8%. This means that using the criteria of at least one food additive with sole cosmetic function, or a substance of rare culinary use to identify UPFs, resulted in high sensitivity, while maintaining an acceptable level of specificity.

Finally, sensitivity analysis further tested the inclusion of vitamins and minerals, which can serve dual purposes, such as fortification or cosmetic enhancement, with the label not clearly indicating whether the primary intent is fortification or cosmetic function. The analysis revealed no significant differences in UPF classification when these components were added to scenarios 2 and 3, suggesting that these multifunctional additives do not substantially influence the identification process.

The present study is not free of limitations that should be considered in the interpretation of the findings. First, we used the information declared in the food package to identify markers of UPFs. The use of the list of ingredients poses some difficulties. For instance, functional classes of food additives are not always displayed, and many additives may have more than one function (34). To address this, we proposed different scenarios, including those that identify food additives with a sole cosmetic function. The presence of compound ingredients (such as chocolate, cookies, cheese, etc.) also represents a challenge and potentially leads to an underestimation of the presence of both food substances and cosmetic additives in food products. Another limitation is the use of 2017 food label data; however, as the study focused on testing methods for identifying UPFs, the dataset remains suitable despite possible product changes over time. Finally, while local food regulations and labeling practices should be considered when applying the scenarios in other contexts, some markers of ultra-processing, such as ingredients of rare culinary use, are widely recognized across countries (23). Additionally, we used food additives listed in the Codex Alimentarius (29), an internationally recognized standard that is updated annually, and these updates should be taken into account when applying or adapting the approach.

This study’s key strengths include the use of a large and representative database (2017 Brazilian Food Labels Database), which provides detailed ingredient information for nearly 10,000 food products. The study employs an extensive list of substances and food additives from the Codex Alimentarius, enabling a detailed and systematic classification of UPFs. By proposing five distinct scenarios, the study evaluates different methods for UPFs identification. The study offers a standardized and replicable methodology that has practical implications for research and public health policies targeting UPFs. Furthermore, considering that the UPF classification is a technical and socially constructed concept (2), this work contributes to advancing methodological clarity while also creating space for further discussions, to explore how such classifications are interpreted and applied by different stakeholders.

In conclusion, the results showed that the use of substances of rare culinary along with food additives with sole cosmetic function is a potentially replicable method to identify UPFs. This approach can helps different stakeholders, such as researchers, advocates, and regulatory measures to inform the designing of policies that require precise definitions of UPFs. It also emphasizes the importance of considering the diverse nature of food categories, as certain products may require tailored criteria for accurate classification according to the Nova food system.

## Data Availability

The original contributions presented in the study are included in the article/Supplementary material, further inquiries can be directed to the corresponding author.
