# Correction: Applying the Nova food classification to food product databases using discriminative ingredients: a methodological proposal

**DOI:** 10.3389/fpubh.2025.1665972

**Published:** 2025-08-04

**Authors:** Mariana Fagundes Grilo, Beatriz Silva Nunes, Ana Clara Duran, Camila Zancheta Ricardo, Larissa Galastri Baraldi, Euridice Martinez Steele, Camila Aparecida Borges

**Affiliations:** ^1^Center for Food Studies and Research, University of Campinas, Campinas, Brazil; ^2^Department of Exercise and Nutrition Sciences, The George Washington University, Washington, DC, United States; ^3^Graduate Program in Collective Health, Faculty of Medical Sciences, University of Campinas, Campinas, Brazil; ^4^Center for Epidemiological Studies in Nutrition and Health, University of São Paulo, São Paulo, Brazil; ^5^Center for Research in Food Environments and Prevention of Chronic Diseases Associated With Nutrition (CIAPEC), Institute of Nutrition and Food Technology (INTA), University of Chile, Santiago, Chile; ^6^Department of Food Science and Technology, University of São Paulo, São Paulo, Brazil

**Keywords:** ultra-processed foods, methodology, classification, food substances, food additives

Author [Beatriz Silva Nunes] was erroneously spelled as [Beatriz Nunes].

Citation [Grilo MF] was erroneously spelled as [Fagundes Grilo M].

Incorrect version of citation: Fagundes Grilo M, Nunes B, Duran AC, Zancheta Ricardo C, Baraldi LG, Martinez Steele E and Borges CA (2025) Applying the Nova food classification to food product databases using discriminative ingredients: a methodological proposal. *Front. Public Health* 13:1575136. doi: 10.3389/fpubh.2025.1575136

Correct version of citation: Grilo MF, Nunes BS, Duran AC, Zancheta Ricardo C, Baraldi LG, Martinez Steele E and Borges CA (2025) Applying the Nova food classification to food product databases using discriminative ingredients: a methodological proposal. *Front. Public Health* 13:1575136. doi: 10.3389/fpubh.2025.1575136

The Author contribution statement should read:

